# Apolipoprotein E4 Frequencies in a Japanese Population with Alzheimer's Disease and Dementia with Lewy Bodies

**DOI:** 10.1371/journal.pone.0018569

**Published:** 2011-04-28

**Authors:** Seiju Kobayashi, Masaru Tateno, Tae Woo Park, Kumiko Utsumi, Hitoshi Sohma, Yoichi M. Ito, Yasuo Kokai, Toshikazu Saito

**Affiliations:** 1 Department of Neuropsychiatry, Sapporo Medical University School of Medicine, Sapporo, Japan; 2 Department of Psychiatry, Sunagawa City Medical Center, Sunagawa, Japan; 3 Department of Educational Development, Sapporo Medical University Center for Medical Education, Sapporo, Japan; 4 Hokkaido Organization for Translational Research, Hokkaido University Graduate School of Medicine, Sapporo, Japan; 5 Department of Biomedical Engineering, Sapporo Medical University School of Medicine, Sapporo, Japan; University Medical Center Groningen, University of Groningen, Netherlands

## Abstract

**Background:**

The apolipoprotein E (APOE) ε4 allele has been reported to be a risk factor for Alzheimer's disease (AD) and dementia with Lewy bodies (DLB). Previous neuropathological studies have demonstrated similar frequencies of the APOE ε4 allele in AD and DLB. However, the few ante-mortem studies on APOE allele frequencies in DLB have shown lower frequencies than post-mortem studies. One reason for this may be inaccuracy of diagnosis. We examined APOE genotypes in subjects with AD, DLB, and a control group using the latest diagnostic criteria and MRI, SPECT, and MIBG myocardial scintigraphy.

**Methods:**

The subjects of this study consisted of 145 patients with probable AD, 50 subjects with probable DLB, and a control group. AD subjects were divided into two groups based on age of onset: early onset AD (EOAD) and late onset AD (LOAD). All subjects had characteristic features on MRI, SPECT, and/or myocardial scintigraphy.

**Results:**

The rate of APOE4 carrier status was 18.3% and the frequency of the ε4 allele was 9.7% in controls. The rate of APOE4 carrier status and the frequency of the ε4 allele were 47% and 27% for LOAD, 50% and 31% for EOAD, and 42% and 31% for DLB, respectively.

**Conclusion:**

The APOE4 genotypes in this study are consistent with previous neuropathological studies suggesting accurate diagnosis of AD and DLB. APOE4 genotypes were similar in AD and DLB, giving further evidence that the ε4 allele is a risk factor for both disorders.

## Introduction

The apolipoprotein E (APOE) ε4 allele has been reported to be a risk factor for Alzheimer's disease (AD) [1], [2]. APOE is a major component of lipoproteins and plays a role in the metabolism and redistribution of cholesterol [3]. APOE levels increase after brain injury in some neurons and APOE can affect neurite extension. Thus, it is thought to play an important role in the repair and protection of neurons [4]. APOE exists as 3 major alleles (ε2, ε3, and ε4) that translate into three isoforms of the protein (APO E2, E3, and E4). Persons being homozygous for the ε4 allele are more likely to develop the sporadic type of AD [5].

APOE4 has also been implicated in the development of dementia with Lewy bodies (DLB), thought to be the second most common cause of dementia [6]. Previous neuropathological studies have demonstrated that the frequency of the APOE ε4 allele in DLB is similar to AD [7], [8], [9], [10], suggesting a common pathophysiology in the two disorders. However, the few antemortem studies on APOE allele frequencies in DLB have shown lower rates of APOE allele frequencies. One reason may be low accuracy of DLB diagnosis. Previous antemortem studies have not used the most current consensus diagnostic criteria for DLB delineated by the DLB consortium in 2005 [11]. Additionally, radiological testing such as MIBG myocardial scintigraphy was not used for diagnosis.

In this study, we examined APOE genotypes in a population of Japanese subjects diagnosed using relevant radiological testing and the most current consensus diagnostic criteria for DLB and AD.

## Materials and Methods

### 1. Subjects (Table 1)

**Table 1:** Subject background.

	Controls	late onset AD	early onset AD	DLB
n	279	129	16	50
Sex (M∶F)	113∶166	44∶85	7∶9	15∶35
Age (y)	75.6±8.1	79.1±4.9	63.1±5.0	79.1±4.9
Age at onset (y)		75.6±5.2	59.3±5.9	75.5±5.0
MMSE score		15.5±5.5	15.8±7.2	15.3±5.4
Period of education (y)		9.1±2.5	11.2±1.7	8.6±3.0

The subjects of this study were 279 control subjects from one of our previous studies [12], 145 patients with probable AD according to the NINCDS/ADRDA criteria [13], and 50 subjects with probable DLB according to the latest consensus diagnostic criteria published in 2005 [11]. To obtain non-demented controls, elderly individuals were recruited in Kitahiroshima, Japan and were evaluated by a questionnaire that included an inquiry into past and present illnesses. These population-based non-demented controls consisted of 113 men and 166 women with a mean±SD age at blood draw of 75.6±8.1 years. All AD subjects had characteristic neuroimaging features such as atrophy of the parahippocampal gyrus and the parietal lobe on MRI and hypoperfusion in the regions beside the posterior cingulate gyrus on 99mTc-ECD brain perfusion SPECT. They were divided into two groups based on the age of onset; early onset AD (EOAD, <65 y) and late onset AD (LOAD, 65 y and higher). Age of onset was determined by interview of patient families. The EOAD group included 16 patients whose mean age was 63.1±5.0 years and male∶female ratio was 7∶9, while the LOAD group consisted of 129 patients whose mean age was 79.1±4.9 years and M∶F ratio was 44∶85. The DLB group consisted of 50 patients whose mean age was 79.1±4.9 years and M∶F ratio was 15∶35. All DLB subjects had preserved hippocampal volume on MRI demonstrated by an automated volume measuring program named VARSD [14], occipital hypoperfusion on brain perfusion SPECT revealed by statistical analysis programs [15], [16], [17], [18], [19], [20], [21] and decreased cardiac uptake of 123I- metaiodobenzylguanidine (MIBG) defined as delayed heart-to-mediastinum (H/M) ratio lower than 1.83 [17], [18], [22].

The APOE genotyping was performed using the polymerase chain reaction. Informed written consent was obtained from all subjects and their relatives. This study was approved by the institutional ethical committees at Sapporo Medical University.

### 2. Method

#### APOE genotyping (Figure 1)

**Figure 1:**
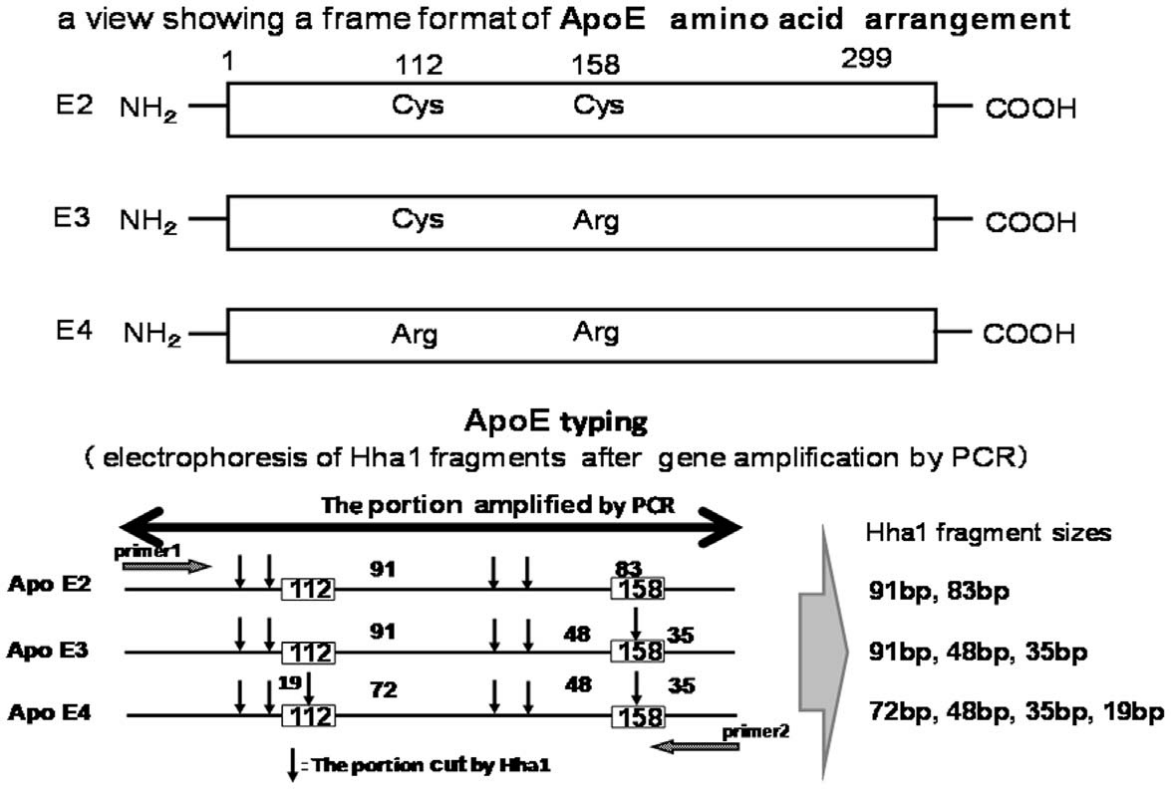
ApoE isoform and Hha1cleavage maps. Each genotype of apoE was distinguished by unique combinations of Hha1 fragments. Cys:Cysteine, Arg:Arginine.

Using a QIAamp DNA Blood Mini Kit (QIAGEN, Tokyo, Japan), genomic DNA was extracted from the buffy coat after centrifugation of the blood sample (1 ml) according to the manufacture's instructions. DNA genotyping of APOE was performed according to the protocol described by Hixson and Vernier et al [23]. Briefly, leukocyte DNA was amplified by PCR using the oligonucleotide primers Primer 1 (5′-TAAGCTTGGCACGGCTGTCCAAGGA-3′) and Primer 2 (5′-ACAGAATTCGCCCCGGCCTGGTACAC-3′) set on common sequence parts of APOE isoforms. The PCR products were digested with Hha1 (New England Biolabs. Japan Inc., Tokyo, Japan) and the resulting digestion fragments were separated by electrophoresis on polyacrylamide gels (SuperSepTMDNA 15% gel(Wako, Tokyo, Japan)). Each genotype of APOE was distinguished by unique combinations of Hha1 fragment sizes in all homozygotic and heterozygotic combinations [23]. After determining the APOE genotypes, we investigated the APOE4 carrier status and the frequency of the ε4 allele in the 279 controls, 145 AD, and 50 DLB cases.

#### Statictical Analysis (Table 2)

**Table 2:** ApoE4 carrier status and the frequency of the ε4 allele in Controls, LOAD, EOAD, and DLB.

	ε2/2	ε2/3	ε3/3	ε2/4	ε3/4	ε4/4	E4 carrier	ε4 allele
Contols N = 279	0(0)	20(7.1)	208(74.6)	2(0.7)	46(16.5)	3(1.1)	18.3%	9.7%
LOAD N = 129	0(0)	5(3.9)	63(48.8)	2(1.5)	50(38.8)	9(7.0)	47.3%	27.1%
EOAD N = 16	0(0)	0(0)	8(50.0)	1(6.2)	5(31.3)	2(12.5)	50.0%	31.3%
DLB N = 50	0(0)	5(10.0)	24(48.0)	2(4.0)	16(32.0)	3(6.0)	42.0%	24.0%

n (%).

Differences in APOE carrier status between groups were evaluated by the Cochran-Mantel-Haenszel Test. It was used to test if there is a relationship between patients (LOAD, EOAD, and DLB) and controls after blocking across a third classification: Wild, Hetero (heterozygous), and Homo (homozygous).That is to say, these comparisons was performed in 2×3 contingency tables. The frequencies of the ε4 allele between groups were compared in 2×2 contingency tables using the Fisher's Exact Test. All analyses were conducted with JMP version 7 (SAS Institute Inc., Cary, NC).

## Results

The results are shown in Table 2.

In the control group, 51 out of 279 subjects were APOE4 carriers (18.3%). Three subjects were homozygous for the ε4 allele (1.1%) and 48 subjects were heterozygous for the ε4 allele (17.2%).The total frequency of the ε4 allele was 9.7%.

In the LOAD group, 61 out of 129 subjects were APOE4 carriers (47.3%). Nine subjects were homozygous for the ε4 allele (7.0%) and 52 subjects were heterozygous for the ε4 allele (40.3%). The total frequency of the ε4 allele was 27.1%.

In the EOAD group, 8 out of 16 subjects were APOE4 carriers (50%). Two subjects were homozygous for the ε4 allele (12.5%) and 6 subjects were heterozygous for the ε4 allele (37.5%). The total frequency of the ε4 allele for this group was 31.3%.

In the DLB group, 21 out of 50 subjects were APOE4 carriers (42%). Three subjects were homozygous for the ε4 allele (6.0%) and 18 subjects were heterozygous for the ε4 allele (36%).The total frequency of the ε4 allele was 24%.

APOE carrier status was significantly different between LOAD and controls (*p<0.0001*), between EOAD and controls (*p = 0.0002*), and between DLB and controls (*p = 0.0003*). Allele frequencies of APOE ε4 were significantly higher in LOAD (*p<0.0001*), EOAD (*p = 0.0011*), and DLB (*p<0.0002*) than in controls (Table 2). There were no significant differences in rates of APOE4 carrier status (*p = 0.82*) and the frequencies of the ε4 allele (*p = 0.59*) between LOAD and DLB. No significant differences in rates of APOE4 carrier status (*p = 0.66*) and the frequencies of the ε4 allele (*p = 0.49*) were found between EOAD and DLB. Similarly, no significant differences were found in rates of APOE4 carrier status (*p = 0.73*) and the frequencies of the ε4 allele (*p = 0.68*) between LOAD and EOAD.

## Discussion

This study examined the rate of APOE4 carrier status and the frequency of the ε4 allele in a group of non-demented subjects, subjects with probable AD who were separated into early-onset and late-onset groups, and subjects with DLB. A significant difference in the distribution of APOE phenotypes was found between each patient group (LOAD, EOAD, and DLB) and controls. We found no significant difference in rates of APOE4 carrier status and the frequencies of the ε4 allele between LOAD/EOAD and DLB subjects. The APOE genotypes in our control subjects are similar to those reported in control subjects from other Japanese studies [24], [25].

Our study results amongst the AD and DLB patients are similar to previous postmortem studies examining APOE4 carrier status and ε4 allele frequencies. In postmortem studies examining LOAD, the frequency of APOE4 carrier status ranges from 47% to 59%, and that of the ε4 allele ranges from 27% to 37% [7], [8]. Regarding antemortem studies on LOAD, the frequency of APOE4 carrier status is 40% to 59%, and that of the ε4 allele is 24 to 31% [24], [26], [27]. Although there are only a few autopsy-based studies that measure APOE4 in EOAD [7], [10], the frequency of APOE4 carrier and ε4 allele was 52%–57% and around 35%, respectively, in those studies. Previous antemortem studies examining EOAD have been widely divergent. Okuizumi et al. investigated 33 antemortem AD cases and 11 postmortem cases and reported that the frequency of APOE4 carrier and ε4 allele is 58% and 30%, respectively [28]. The frequency of APOE4 carrier status and the ε4 allele in EOAD was clinically reported as 7% and 4% by Kawamata et al. [29], and 72% and 43% by Dai et al [30], respectively. The widely divergent results suggest that antemortem studies, particularly involving EOAD, might depend strongly on methodological differences. Our results were consistent with previous postmortem studies in both LOAD and EOAD, supporting the accuracy of our diagnoses.

Although there are many clinical [9], [30] and autopsy-based studies [31], [32] measuring the ε4 allele in AD, most do not distinguish between EOAD and LOAD. However, both have different genetic backgrounds [33], different rates of progression [34], and different levels of impairment in verbal and visual cognition [35], [36]. Thus, it is necessary to distinguish EOAD from LOAD for research purposes. The NINCDS/ADRDA criteria support this notion and states that researchers should be aware of two subtypes; “less than 65 y” and “65 y and higher” [13].

With regards to DLB, our results also closely matched postmortem studies examining the frequencies of APOE4 carrier status and the ε4 allele. Several research groups from various countries have conducted similar investigations with large samples of postmortem brains of DLB patients [7], [9], [10]. The frequency of APOE4 carrier status and the ε4 allele in these studies ranged from 36% to 65% and 19 to 36%, respectively. There have been three previous antemortem studies that have reported the frequency of APOE4 carrier status and the ε4 allele in DLB patients. Engelborghs et al. [32] reported the frequency of APOE4 carrier status and the ε4 allele as 28.5% and 18%, respectively, Carrillo et al. [31] indicated frequencies of 27.6% and 16.4%, and Lane et al. [37] reported frequencies of 35% and 18.1%. These frequencies are lower or at the lower end of the range of frequencies determined by autopsy-based studies, as opposed to our results which were more consistent with previous autopsy-based studies.

Accurate antemortem diagnosis of DLB is important. Some patients with DLB may have an accelerated disease progression and approximately 50% of patients experience life-threatening adverse reactions to antipsychotic medications [38]. Our results suggest that our clinical diagnosis of DLB and AD was quite accurate. To accomplish the highest possible accuracy of antemortem diagnosis of DLB, we performed MRI, brain perfusion SPECT and MIBG myocardial scintigraphy in conjunction with using the latest DLB diagnostic criteria. These diagnostic guidelines [11] acknowledge the importance of the types of neuroimaging findings found in our study such as relative preservation of medial temporal lobe structures on MRI scan, occipital hypoperfusion on brain perfusion SPECT and decreased MIBG uptake on myocardial scintigraphy. We have previously demonstrated the usefulness of these neuroimaging tools for the clinical diagnosis of DLB [18], [22]. Notably, all of the previous antemortem studies [31], [32], [37] on APOE4 in DLB did not use or were conducted before the publication of the current consensus diagnostic criteria for DLB. Our study is the first antemortem study to use the latest diagnostic criteria for DLB. The results of our study suggest that the current criteria used in conjunction with MIBG myocardial scintigraphy, MRI and PET is more accurate in the diagnosis of DLB than previous diagnostic criteria.

There is a growing consensus that APOE allele frequency is influenced by race [39]. This may explain why the frequency of the ε4 allele in autopsied DLB cases varies widely (19 to 36%). To the best of our knowledge, there are no antemortem studies in which APOE4 genotypes are characterized in Asian patients with DLB. A prior meta-analysis found that the APOE ε4 allele association with AD was stronger in Japanese subjects than in Caucasian subjects [39]. Our results suggest that the APOE ε4 allele is more frequent in Japanese patients with DLB.

The role of APOE ε4 in the pathophysiology of AD remains controversial. However, it has been reported that the gene dose of APOE ε4 correlates with the expression of senile plaques (SP) and neurofibrillary tangles (NFT) [2]. SPs are composed of amyloid-beta protein (Aβ) which is deposited outside the neuron. Previous studies have revealed a large quantity of Aβ deposition in the brain of AD patients with APOE4 [40]. Aβ deposition has been shown in the neuropil and vessel walls of subjects with APOE4 [40]. Notably, the extent of Aβ deposition in APOE4 homozygotes was more severe than APOE4 heterozygotes [40]. On the other hand, NFTs are composed of phosphorylated tau proteins that have accumulated inside the neuron. Animal studies have revealed that transgenic mice expressing human APOE4 had more phosphorylated tau proteins [26].

Our results demonstrated similar frequencies of APOE4 carrier status and the ε4 allele in LOAD, EOAD and DLB. Indeed, previous studies have revealed the commonality of pathological findings in DLB and AD. Kosaka reported that many SPs and/or NFTs are present in autopsy cases with DLB. According to his report, DLB can be divided into two forms: a common form and a pure form [41]. In the common form, numerous Lewy bodies can be found with many SPs and/or NFTs in the cerebral cortex, whereas in the pure form, there are few or no senile changes. Kawanishi et al. also reported that SPs, a characteristic feature of AD, were found in the autopsied brains of DLB patients [27]. We speculate that DLB patients with AD pathology tend to have higher frequencies of the ε4 allele. Perhaps a higher portion of our DLB patient sample have AD pathology, though due to the antemortem nature of our study it is difficult to assess this.Given the similar frequencies of ε4 allele in AD and DLB in our study, our results support the idea that there is often overlapping pathology between AD and DLB and that APOE4 plays a role in the pathophysiology of both disorders.

We must recognize some limitations in our study. The number of subjects in our study was small, although we included 50 probable DLB cases, a number higher than previous studies. Also we have not confirmed the diagnosis through neuropathological means. Current guidelines suggest pathological confirmation to give a definite diagnosis of DLB. However, our results suggest that the combination of careful history taking, conscientious examination of clinical features, neuro-psychological cognitive tests, and neuroimaging tools including MRI, brain perfusion SPECT and MIBG myocardial scintigraphy could significantly increase the preciseness of clinical diagnosis.

In conclusion, our study results give further support for APOE4 as a biological marker for the presence of DLB. Our results demonstrated that the rate of APOE carrier status and the frequency of the ε4 allele in DLB were as high as in AD. Our results were also consistent with postmortem studies suggesting that our diagnosis including brain perfusion SPECT and MIBG myocardial scintigraphy was accurate.
